# Supplementary material for: Mechanism of membrane pore formation by human gasdermin‐D
Source: EMBO J. 2018 Jun 13;37(14):e98321. doi: 10.15252/embj.201798321 (PMC6043855; doi:10.15252/embj.201798321)
Supplement: Supplementary file 1 — Appendix [file EMBJ-37-e98321-s001.pdf]

Appendix

Appendix Table of Contents

Appendix Figure S1. Characterization of the supported lipid membrane (SLM) by AFM imaging.

Appendix Figure S2. GSDMD is cleaved by three different caspases to the same fragments.

Appendix Figure S3. Additional time-lapse AFM of GSDMD^{Nterm} oligomerization and pore formation.

Appendix Figure S4. Additional time-lapse AFM of GSDMD^{Nterm} oligomerization and pore formation.

Appendix Figure S5. Time-lapse topographs of GSDMD^{Nterm} oligomerization and pore formation in SLMs made from POPG and POPC.

Appendix Table S1. Characterization of slit- and ring-shaped GSDMD^{Nterm} oligomers formed in SLM made from different lipid compositions and imaged by AFM.

Appendix Table S2. Characterization of slit- and ring-shaped GSDMD^{Nterm} oligomers imaged by AFM after cleavage of GSDMD by either caspase-1, -4 or -5.

Appendix Table S3. Summary of lipid membrane compositions tested to characterize GSDMD^{Nterm} binding, oligomerization and pore formation.

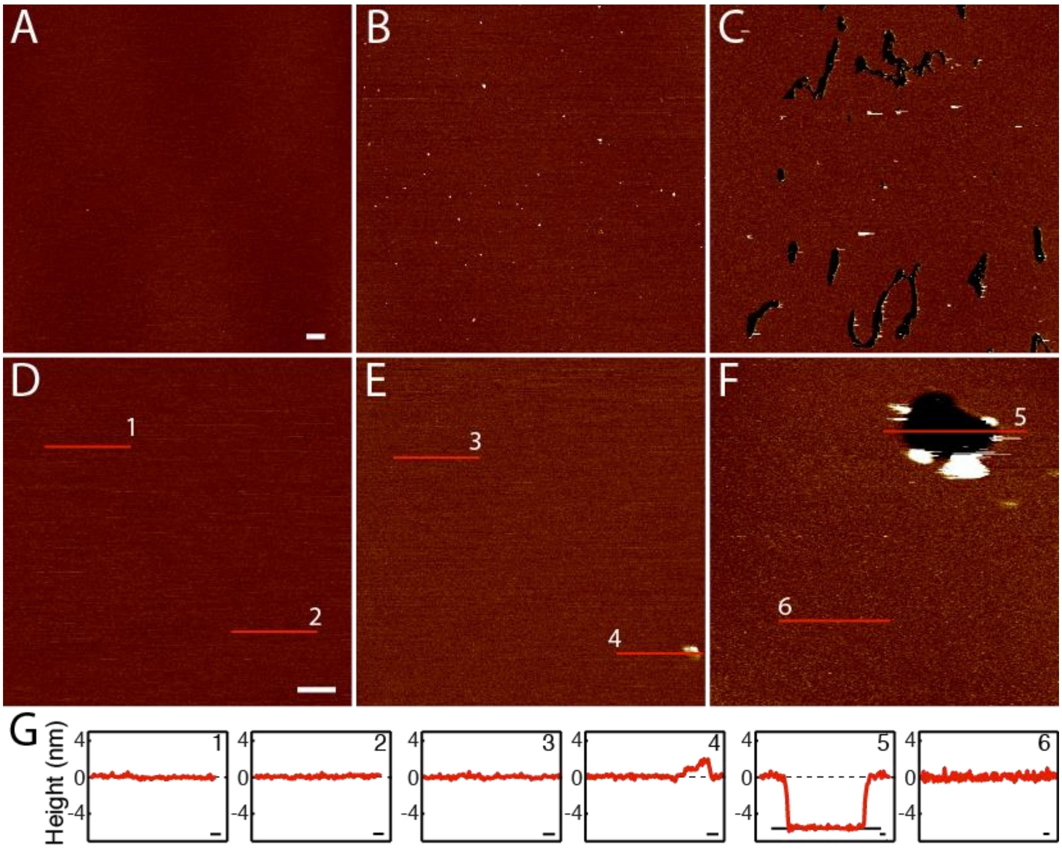

Appendix Figure S1. Characterization of the supported lipid membrane (SLM) by AFM imaging.

(A) AFM topograph of freshly cleaved atomically flat muscovite mica.

(B) Topograph of SLM made from *E. coli* polar lipid extract and uniformly covering the supporting mica.

(C) Topograph recorded of SLM made from *E. coli* polar lipid extract after air drying for 1 min. Holes and cracks of the SLM (black areas) are observed.

(D, E) Topographs of SLM made from *E. coli* polar lipid extract and uniformly covering the supporting mica.

(F) Topograph recorded of SLM made from *E. coli* polar lipid extract after air drying for 1 min. Holes and cracks of the SLM (black areas) are observed.

(G) Height profiles measured along the red lines indicated in AFM topographs (D-F). The black thick line in (5) indicates the mica surface at ≈ –5 nm height and the black dashed lines indicate the SLM surface at ≈ 0 nm height in (4, 5).

All AFM topographs were recorded in buffer solution at room temperature (Materials and Methods). The full color range of the topographs corresponds to a vertical scale of 5 nm. Scale bars, 1 µm (A-C) and 50 nm (D-F).

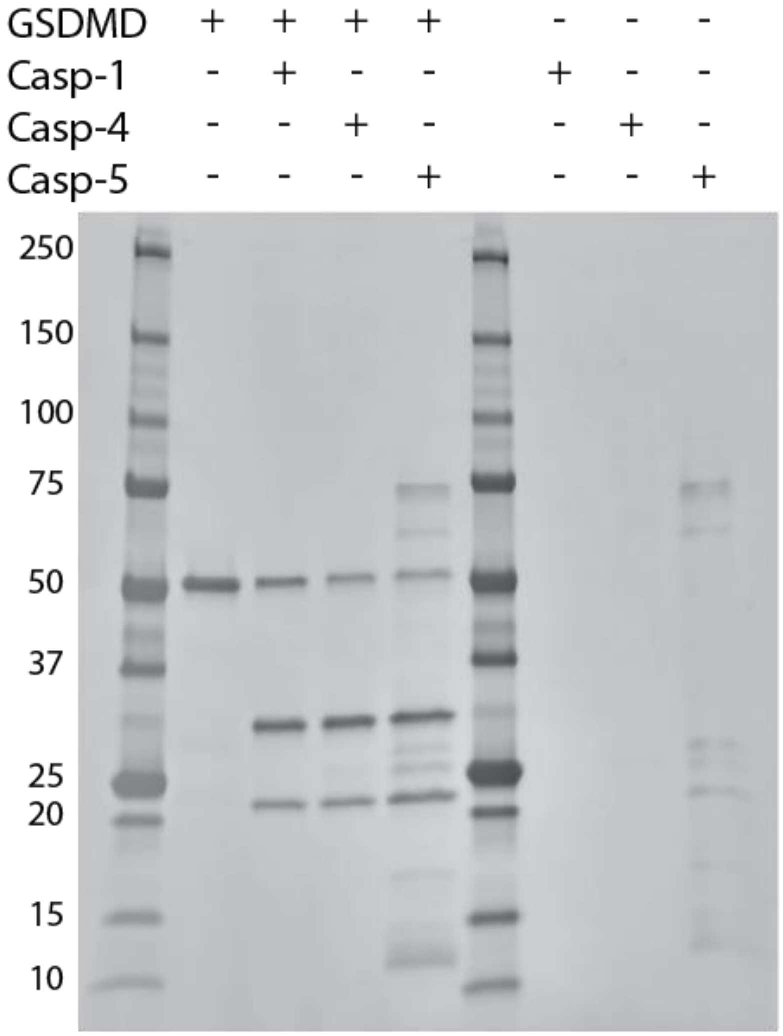

Appendix Figure S2. GSDMD is cleaved by three different caspases to the same fragments.

SDS-PAGE analysis of a protease cleavage reaction of 2 µM human GSDMD cleaved with either 5 nM caspase-1, -4, or -5 (Casp-1, Casp-4, Casp-5). Cleavage of the 53 kDa large GSDMD results in two fragments of 31 kDa ($GSDMD^{Nterm}$) and 22 kDa ($GSDMD^{Cterm}$). Molecular weight markers (in kDa) are annotated. Note that the caspase-5 sample contains trace impurities, which do not affect the cleavage reaction.

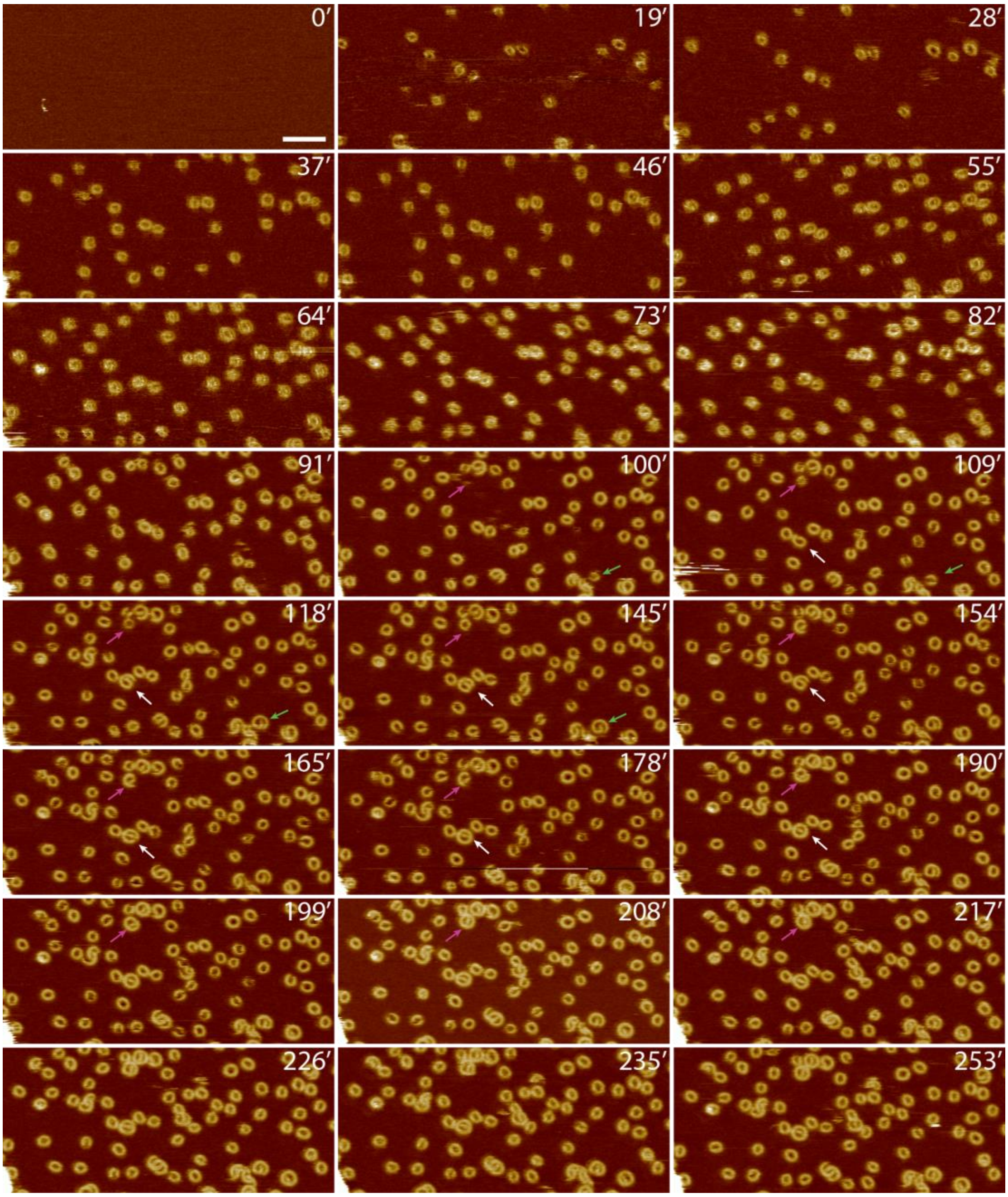

Appendix Figure S3. Additional time-lapse AFM of GSDMD^{Nterm} oligomerization and pore formation.

The first FD-based AFM topograph of a SLM made from POPC, DOPG, DOPS, DOPE, and CL (40:20:10:20:10 molar ratio) was used as a control to show that the lipid membrane was defect-free. The defect-free SLM was then incubated with GSDMD and caspase-1 in buffer solution at 37°C. Recorded at different time points of the incubation (indicated by time stamps in min) the time-lapse topographs follow the progress of GSDMD^{Nterm} binding and assembly to the SLM. Arrows indicate examples of GSDMD^{Nterm} arc- or slit-shaped oligomers that grow into larger ring-shaped oligomers. The full color range of the topographs corresponds to a vertical scale of 10 nm. Scale bar, 50 nm.

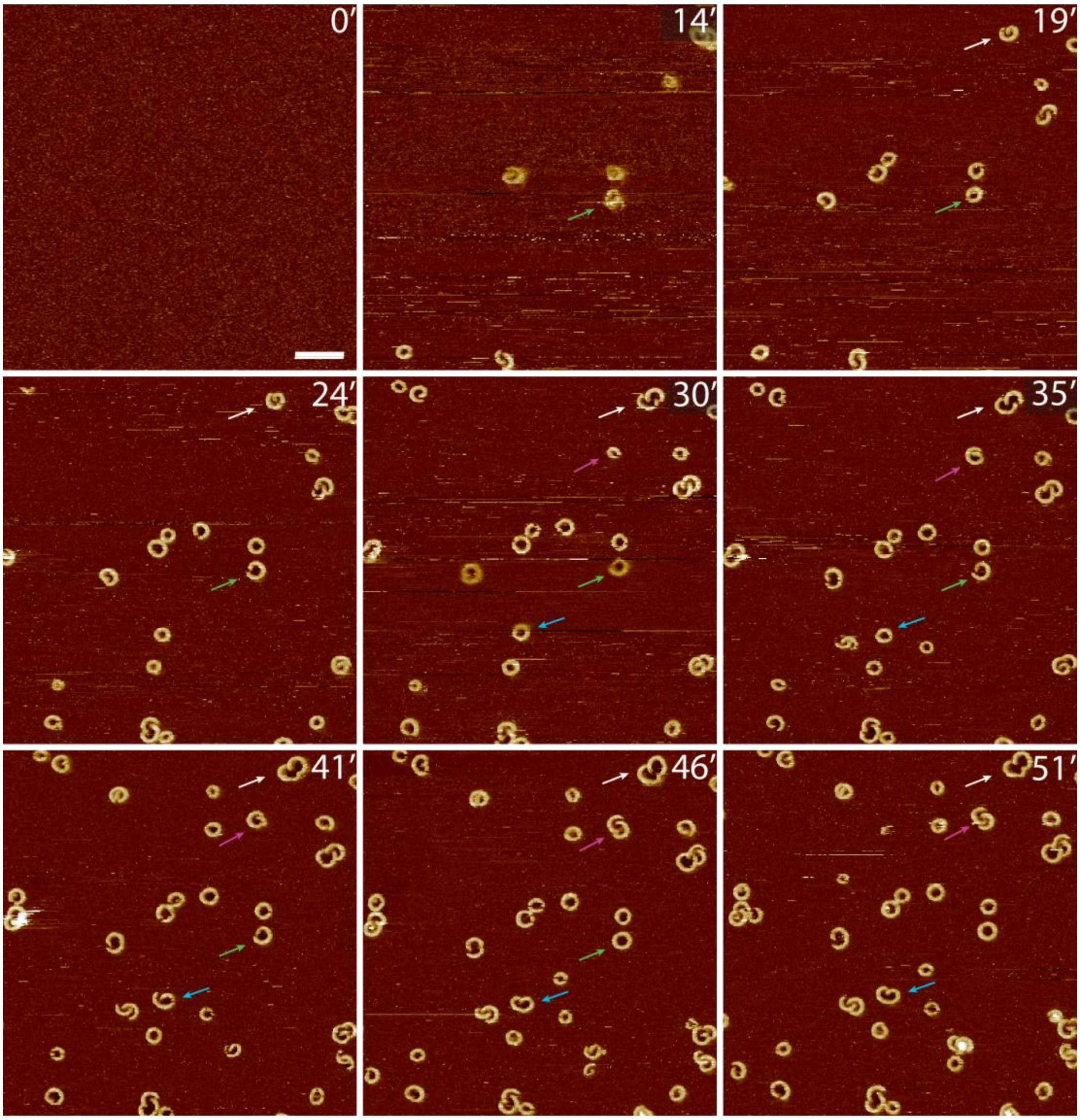

Appendix Figure S4. Additional time-lapse AFM of GSDMD^{Nterm} oligomerization and pore formation.

The first FD-based AFM topograph of a SLM made from POPC, DOPG, DOPS, DOPE, and CL (40:20:10:20:10 molar ratio) was used as a control to show that the SLM was defect-free. The defect-free SLM was then incubated with GSDMD and caspase-1 in buffer solution at 37°C. Recorded at different time points of the incubation (indicated by time stamps in min) the time-lapse topographs follow the progress of GSDMD^{Nterm} binding and assembly to the SLM. Arrows indicate examples of GSDMD^{Nterm} oligomers growing into larger oligomers. The full color range of the topographs corresponds to a vertical scale of 10 nm. Scale bar, 100 nm.

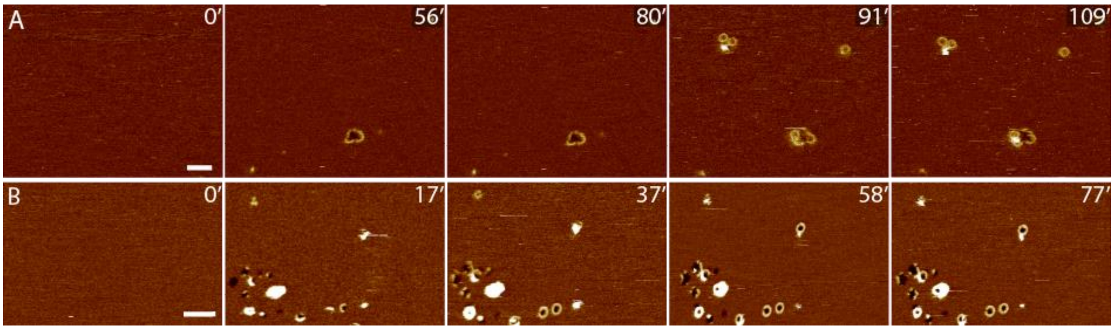

Appendix Figure S5. Time-lapse topographs of GSDMD^{Nterm} oligomerization and pore formation in SLMs made from POPG and POPC.

The first AFM topographs at the left show SLMs made from (A) POPG or (B) POPC before of their incubation with GSDMD and caspase-1. The following AFM topographs show the SLMs incubated with GSDMD and caspase-1 buffer solution at 37°C. In each topograph the time point of the incubation is indicated by the time stamp (in min). The time-lapse FD-based AFM topographs were recorded in buffer solution at 37°C as described (**Materials and Methods**). The full color range of the topographs corresponds to a vertical scale of 10 nm. Scale bars, 50 nm

SLM	Slit-shaped oligomers Height above SLM (mean ± SE)	Ring-shaped oligomers Height above SLM (mean ± SE)	Ring-shaped oligomers Diameter (mean ± SE)
POPC, DOPG, DOPS, DOPE, and CL (40:20:10:20:10 molar ratio)	3.6 ± 0.2 nm<br>(*n* = 74)	3.6 ± 0.3 nm<br>(*n* = 277)	22.6 ± 0.3 nm<br>(*n* = 288)
E. coli polar lipid extract	3.5 ± 0.3 nm*<br>(*n* = 135)<br>*P* = 0.01	3.5 ± 0.3 nm^{NS}<br>(*n* = 164)<br>*P* = 0.08	23.1 ± 0.7 nm^{NS}<br>(*n* = 183)<br>*P* = 0.32
E. coli polar lipid extract and cholesterol (70:30 weight ratio)	3.5 ± 0.3 nm^{NS}<br>(*n* = 35)<br>*P* = 0.09	3.4 ± 0.4 nm*<br>(*n* = 63)<br>*P* = 0.008	21.2 ± 0.4 nm*<br>(*n* = 94)<br>*P* = 0.001
POPS, DOPE and POPC (35:25:40 molar ratio)	3.2 ± 0.2 nm*<br>(*n* = 23)<br>*P* = 1.8 10^{-12}	3.3 ± 0.2 nm*<br>(*n* = 20)<br>*P* = 3.0 10^{-5}	25.8 ± 1.6 nm^{NS}<br>(*n* = 20)<br>*P* = 0.06
POPS, DOPE and PI(4,5)P2 (35:25:40 molar ratio)	3.2 ± 0.3 nm*<br>(*n* = 53)<br>*P* = 3.0 10^{-5}	3.2 ± 0.2 nm*<br>(*n* = 70)<br>*P* = 2.0 10^{-23}	26.7 ± 0.5 nm*<br>(*n* = 112)<br>*P* = 9.0 10^{-5}

Appendix Table S1. Characterization of slit- and ring-shaped GSDMD^{Nterm} oligomers formed in SLM made from different lipid compositions and imaged by AFM.
Statistical significances were calculated using a two-tailed T-test comparing oligomeric heights relative to the POPC, DOPG, DOPS, DOPE, and CL (40:20:10:20:10 molar ratio) condition. Values were considered significant * if $P < 0.05$ and non-significant (*NS*) if $P \geq 0.05$. FD-based AFM imaging was conducted as described in the main manuscript and **Materials and Methods**.

Caspase	Slit-shaped oligomers Height above SLM (mean ± SE)	Ring shaped oligomers Height above SLM (mean ± SE)	Ring shaped oligomers Diameter (mean ± SE)
GSDMD-caspase-1	3.6 ± 0.2 nm<br>(*n* = 74)	3.6 ± 0.3 nm<br>(*n* = 277)	22.6 ± 0.3 nm<br>(*n* = 288)
GSDMD-caspase-4	3.3 ± 0.2 nm*<br>(*n* = 38)<br>$P = 1.0\ 10^{-9}$	3.4 ± 0.3 nm*<br>(*n* = 196)<br>$P = 1.0\ 10^{-11}$	25.1 ± 0.2 nm*<br>(*n*=230)<br>$P = 5.0\ 10^{-11}$
GSDMD-caspase-5	3.5± 0.4 nm^{NS}<br>(*n* = 41)<br>*P* = 0.1	3.5 ± 0.4 nm*<br>(*n* = 115)<br>*P* = 0.02	25.0 ± 0.1 nm*<br>(*n*=169)<br>$P = 1.0\ 10^{-12}$

Appendix Table S2. Characterization of slit- and ring-shaped GSDMD^{Nterm} oligomers imaged by AFM after cleavage of GSDMD by either caspase-1, -4 or -5.
GSDMD^{Nterm} oligomers were imaged in SLMs made from POPC, DOPG, DOPS, DOPE, and CL (40:20:10:20:10 molar ratio). Statistical significances were calculated using a two-tailed T-test comparing oligomeric heights relative to the caspase-1 condition. Values were considered significant * if $P < 0.05$ and non-significant (*NS*) if $P \geq 0.05$. FD-based AFM imaging was conducted as described in the main manuscript and **Materials and Methods**.

Lipid membrane composition	Data shown in	Membrane Binding	Assembly in arc-, slit- and ring-like oligomers	Pore formation
POPC, DOPG, DOPS, DOPE, and CL (40:20:10:20:10 molar ratio)	Fig. 1, Fig. 3, Fig. 4, Fig. 5, Fig. EV5 Appendix Fig. S3, Appendix Fig. S4,	Yes	Yes	Yes
E. coli polar lipid extract	Fig. 1, Fig. EV3	Yes	Yes	Yes
E. coli polar lipid extract and cholesterol (70:30 weight ratio)	Fig. 1	Yes, reduced	Yes	Yes
POPS, DOPE and POPC (35:25:40 molar ratio)	Fig. 2	Yes	Yes	Yes
POPS, DOPE and POPI (35:25:40 molar ratio)	Fig. 2	No	No	No
POPS, DOPE and PI(4,5)P2 (35:25:40 molar ratio)	Fig. 2	Yes	Yes	Yes
POPS, POPC, DOPE and PI(4,5)P2 (35:30:25:10 molar ratio)	Fig. EV4	Yes	Yes	Yes
POPS, POPC, DOPE, PI(4,5)P2 and cholesterol (30:26:21:8:15 molar ratio)	Fig. EV4	Yes, reduced	Yes	Yes
POPS, POPC, DOPE, PI(4,5)P2 and cholesterol (24:21:18:7:30 molar ratio)	Fig. EV4	Largely suppressed	No	No
POPG	Appendix Fig. S5	Yes, reduced	Yes	Yes
POPC	Appendix Fig. S5	Yes, reduced	Yes	Yes

Appendix Table S3. Summary of lipid membrane compositions tested to characterize GSDMD^{Nterm} binding, oligomerization and pore formation.
