# Supplementary material for: Mechanism of membrane pore formation by human gasdermin‐D
Source: EMBO J. 2018 Jun 13;37(14):e98321. doi: 10.15252/embj.201798321 (PMC6043855; doi:10.15252/embj.201798321)
Supplement: Supplementary file 2 — Expanded View Figures PDF [file EMBJ-37-e98321-s002.pdf]

Expanded View Figures

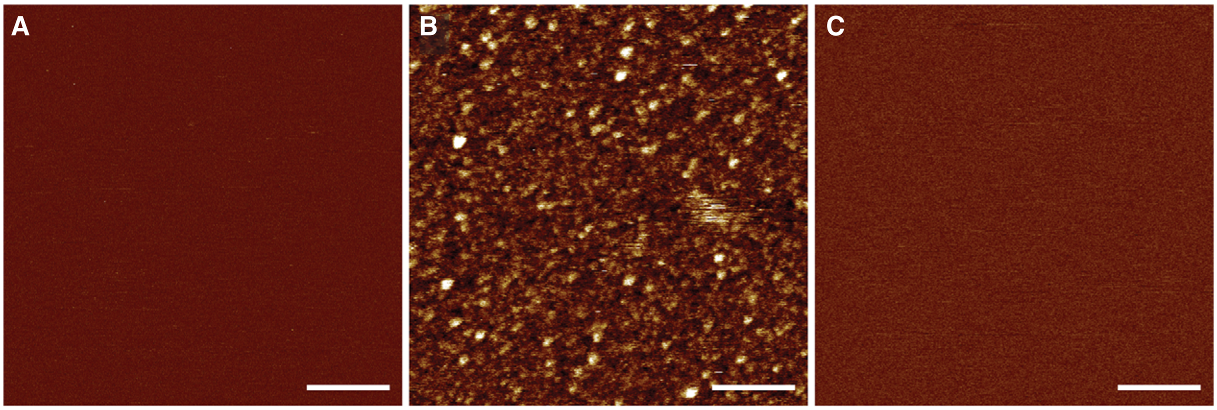

Figure EV1. AFM of human GSDMD incubated with caspase-1 in the absence of a lipid membrane on mica or incubated in the absence of caspase on a SLM.

A AFM topograph of a freshly cleaved atomically flat mica support imaged in buffer solution (50 mM NaCl, 100 mM Hepes, 5 mM TCEP, pH 7.4).

B Topograph of GSDMD (0.5 μM) and caspase-1 (0.1 μM) incubated in buffer solution (50 mM NaCl, 100 mM Hepes, 5 mM TCEP, pH 7.4) for 60 min at 37°C on the mica support.

C Topograph of a SLM made from *E. coli* polar lipid extract and incubated with GSDMD (0.5 μM) in the absence of caspase in buffer solution (50 mM NaCl, 100 mM Hepes, 5 mM TCEP, pH 7.4) for 60 min at 37°C.

Data information: The full color range of the topographs corresponds to a vertical scale of 4.5 nm. Scale bars, 100 nm.

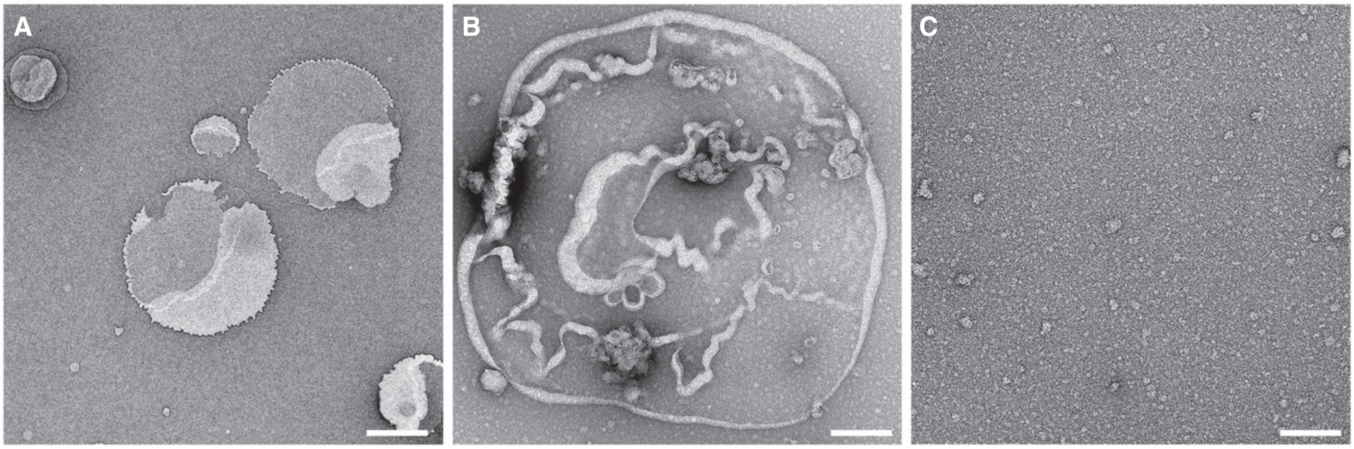

Figure EV2. Transmission electron microscopy (TEM) of empty liposomes, liposomes incubated with GSDMD, and human GSDMD incubated with caspase-1 in the absence of a lipid membrane.

A TEM image of liposomes made from *E. coli* polar lipid extract such as used in this work. During adsorption onto the TEM grid, liposomes fused with each other forming lipid membranes. Liposomes made from other lipids used in this work appeared very similar (not shown here).

B TEM image of liposomes made from *E. coli* polar lipid extract and incubated with 5 μM GSDMD overnight at 37°C in the absence of caspase. None of the lipid membranes imaged ($n > 50$) showed the characteristic arc-, slit-, or ring-like structures observed for GSDMDNterm.

C TEM image of GSDMD (5 μM) incubated with caspase-1 (0.1 μM) overnight at 37°C. No lipid or liposomes has been added to the sample.

Data information: Samples were negatively stained with uranyl acetate and imaged at 120 kV (Materials and Methods). Scale bars, 500 nm (A) and 100 nm (B, C).

© 2018 The Authors

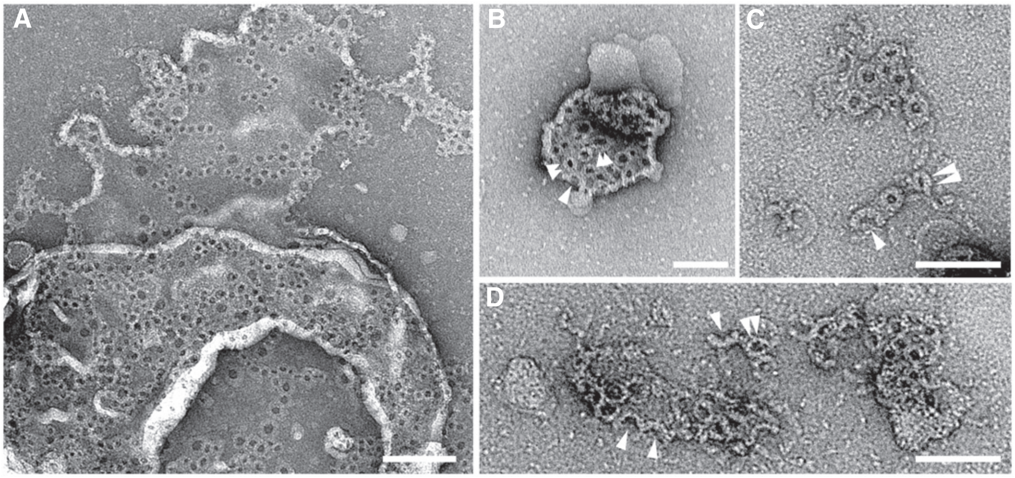

Figure EV3. TEM of arc-, slit-, and ring-shaped GSDMD^{Nterm} oligomers in liposomes.

A, B TEM images of liposomes after having been incubated with GSDMD in the presence of caspase-1. Upon adsorption to the TEM grid most of the liposomes fused with each other forming lipid membranes. GSDMD^{Nterm} oligomers inserted into single layered lipid membranes are observed.

C, D Higher resolution images of arc-, slit-, and ring-shaped oligomers formed by GSDMD^{Nterm}. Arc-like oligomers are pointed out by single arrowheads and slit-like oligomers by double arrowheads.

Data information: Suspended liposomes made from *E. coli* polar lipid extract were incubated overnight at 37°C with GSDMD (5 μM) and catalytic amounts of caspase-1 (0.1 μM). Samples were negatively stained with uranyl acetate and imaged at 120 kV (Materials and Methods). Scale bars, 200 nm (A), 100 nm (B), and 50 nm (C, D).

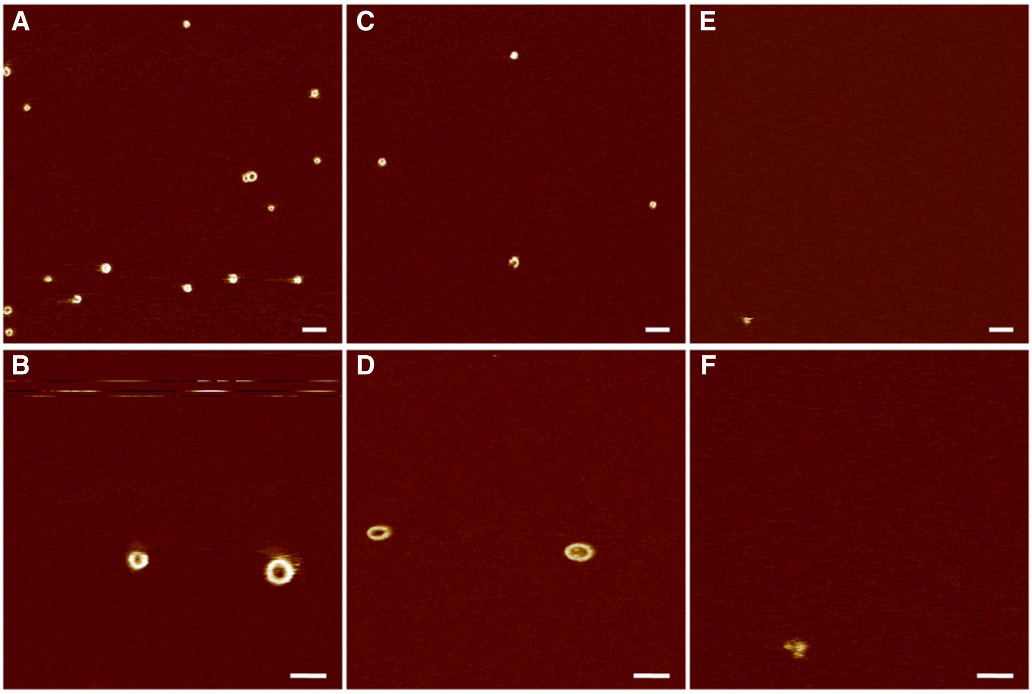

Figure EV4. Effect of cholesterol on the assembly of GSDMD^{Nterm} oligomers in phosphoinositide-containing lipid membranes.

A–F Overview and high-resolution AFM topographs showing GSDMD^{Nterm} oligomers formed on SLMs made from (A, B) POPS, POPC, DOPE, and PI(4,5)P2 (35:30:25:10 molar ratio), (C, D) POPS, POPC, DOPE, PI(4,5)P2, and cholesterol (30:26:21:8:15 molar ratio), and (E, F) POPS, POPC, DOPE, PI(4,5)P2, and cholesterol (24:21:18:7:30 molar ratio). The full color range of the topographs corresponds to a vertical scale of 5 nm. Scale bars, 100 nm (A, C, and E) and 50 nm (B, D, and F).

© 2018 The Authors

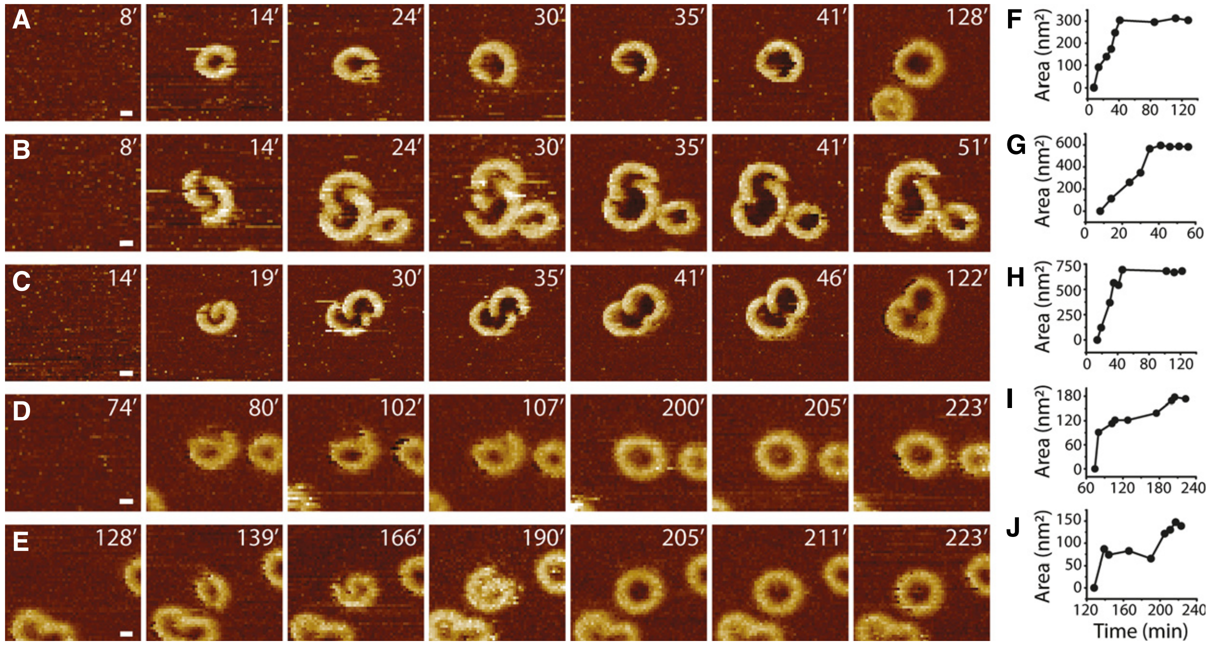

Figure EV5. Analysis of the surface area of the transmembrane pore formed by GSDMD^{Nterm} oligomers over time.

A–E Time-lapse AFM topographs showing the assembly and pore formation of GSDMD^{Nterm} oligomers on SLMs made from POPC, DOPG, DOPS, DOPE, and CL (40:20:10:20:10 molar ratio). Time stamps indicate minutes.

F–J Surface area of transmembrane pores measured in time-lapse AFM topographs. (F) was taken from (A), (G) from (B), (H) from (C), (I) from (D), and (J) from (E).

Data information: Topographs were extracted from time-lapse topographs shown in Fig 4 and Appendix Fig S3 and S4. The full color range of the topographs corresponds to a vertical scale of 7.4 nm. Scale bars, 10 nm.

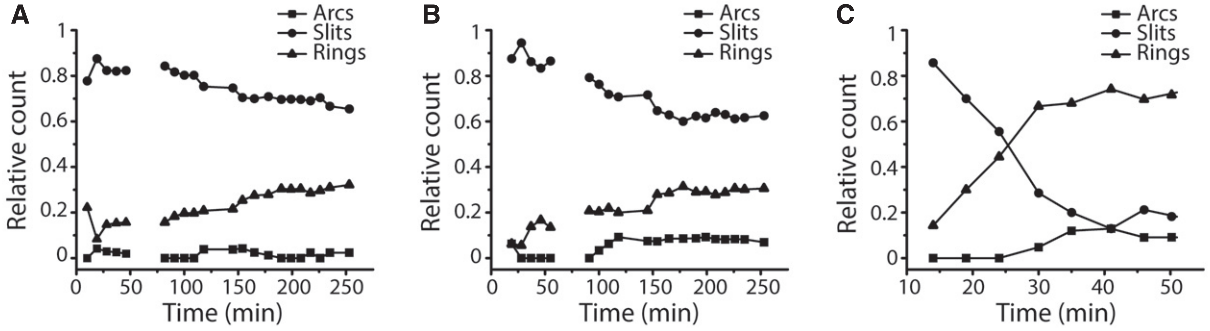

Figure EV6. Relative count of arc-, slit-, and ring-shaped oligomers over time.

A–C The numbers of arc-, slit-, and ring-shaped oligomers were determined from time-lapse AFM topographs recording the GSDMD^{Nterm} oligomerization and pore formation (Fig 4 and Appendix Fig S3 and S4). The number of arc-, slit-, and ring-like oligomers was normalized to 1. The total number of GSDMD oligomers analyzed was 1,398 in (A), 1,142 in (B), and in 178 (C).

 © 2018 The Authors
